# Antioxidant Protein Hydrolysates and Peptides from Catfish: Enzymatic Production, In Vitro Bioactivity, and Translational Gaps for Functional Foods

**DOI:** 10.3390/antiox15050631

**Published:** 2026-05-15

**Authors:** Fai-Chu Wong, Ai-Lin Ooi, Wen-Jie Ng, Fazilah Abd Manan, Tsun-Thai Chai

**Affiliations:** 1 Department of Chemical Science, Faculty of Science, Universiti Tunku Abdul Rahman, Jalan Universiti, Bandar Barat, Kampar 31900, Malaysia; wongfc@utar.edu.my; 2 Center for Agriculture and Food Research, Universiti Tunku Abdul Rahman, Jalan Universiti, Bandar Barat, Kampar 31900, Malaysia; 3 Department of Agricultural and Food Science, Faculty of Science, Universiti Tunku Abdul Rahman, Jalan Universiti, Bandar Barat, Kampar 31900, Malaysia; ooial@utar.edu.my; 4 Department of Allied Health Sciences, Faculty of Science, Universiti Tunku Abdul Rahman, Jalan Universiti, Bandar Barat, Kampar 31900, Malaysia; ngwj@utar.edu.my; 5 Centre for Biomedical and Nutrition Research, Universiti Tunku Abdul Rahman, Jalan Universiti, Bandar Barat, Kampar 31900, Malaysia; 6 Department of Biosciences, Faculty of Science, Universiti Teknologi Malaysia, Skudai 81310, Malaysia; m-fazilah@utm.my

**Keywords:** antioxidant peptide, catfish by-product, enzymatic hydrolysis, functional food, protein hydrolysate, structure–activity relationship

## Abstract

Over the past decade, an increasing demand for natural antioxidants has driven research into antioxidant peptides and protein hydrolysates from fish and their processing by-products. Catfishes, especially species like *Pangasius* and *Clarias*, generate large amounts of protein-rich by-products, which represent a valuable bioresource for valorization. This review discusses advances from the past decade in the production, characterization, and antioxidant capacity of protein hydrolysates and peptides that have been discovered from catfish muscle and by-products. This review emphasizes enzymatic hydrolysis strategies, using Alcalase and other commercial and by-product-derived proteases. Potent antioxidant fractions, particularly those with low molecular weight (<3 kDa) and rich in hydrophobic/aromatic amino acids, have been obtained from the hydrolysates. Mechanisms of antioxidant action, which include hydrogen atom transfer and electron transfer, are discussed in this review, along with the efficacy of catfish-derived antioxidant peptides and protein hydrolysates as demonstrated in chemical and in vivo models. Applications in food systems, such as emulsion-type sausages, have shown potential for shelf-life extension. Nevertheless, knowledge gaps remain, which include an over-dependence on in vitro assays, limited identification of antioxidant peptide sequences, and insufficient data on sensory properties, intestinal permeability, bioavailability, and stability under food processing conditions. Future work should prioritize proteomic characterization, cellular validation, flavor-masking strategies, and scalable production protocols to accelerate the application of catfish protein hydrolysates as viable natural antioxidants for the functional food industry.

## 1. Introduction

In food systems, oxidation of lipids and proteins can give rise to off-flavors, color changes, and nutrient degradation, leading to reduced shelf life and lower consumer acceptance [1,2]. In humans, when the production of reactive oxygen species (ROS) exceeds its antioxidant defenses, oxidative stress occurs. Oxidative stress is a key contributor to aging, inflammation, and certain chronic diseases, including diabetes, neurodegeneration, and cardiovascular disorders [3]. In both cases, antioxidants play an important role in attenuating these effects [4,5]. While the food industry has historically used synthetic antioxidants such as tert-butylhydroquinone, butylated hydroxyanisole, and butylated hydroxytoluene, concerns over health risks and shifting consumer preferences have driven the current search for safer, natural alternatives [6]. This trend has intensified interest in antioxidant peptides and protein hydrolysates, especially those originating from fish muscle and their processing by-products [7,8].

Among various natural sources, fish proteins and their processing by-products are well-recognized as precursors for bioactive peptides with potent antioxidant effects [9,10]. Catfishes, mainly from the genus *Pangasius* and *Pangasianodon* [11,12,13,14,15] but also *Clarias* [16,17], represent a particularly valuable raw material for the exploration of antioxidant peptides and protein hydrolysates. Worldwide, pangasiid aquaculture is one of the fastest-growing whitefish sectors, led by Vietnam. In catfish processing, particularly for *Pangasianodon hypophthalmus*, a substantial proportion of the total fish biomass is generated as by-products such as skin, bone, head, viscera, and trimmings. Such a massive volume of material generated by this sector is often processed into fish meal, or discarded as low-value waste, with improper disposal identified as a source of regional water pollution [18,19]. Notably, these processing by-products are protein-rich (approximately 15% on a wet-weight basis) [20], making them low-cost raw materials for enzymatic hydrolysis to release potent bioactive peptides. Therefore, the valorization of these catfish processing residues is an excellent strategy for developing sustainable, natural antioxidant ingredients for the functional food market.

Over the past decade, numerous studies have reported various antioxidant activities in protein hydrolysates and peptides obtained from catfish muscles and processing by-products using enzymes such as Alcalase, papain, and bromelain [11,14,21]. Yet, despite this growing body of work, the majority of investigations have focused heavily on in vitro chemical assays, with relatively few progressing to peptide sequence identification, stability testing in food matrices, or validation in biological models. Although catfish-derived peptides are often presented as promising functional ingredients, research in this field is still in its early stages. To understand their true potential, a clear distinction must be made between chemical antioxidant activity and actual biological efficacy, which requires the peptide to remain stable and functional after human digestion and intestinal transport. Consequently, although production methods are well-documented, major gaps remain before these peptides can be applied to human health. The near-complete lack of intestinal permeability studies (e.g., Caco-2 models), cellular validation, and bioavailability data highlights the need for a critical synthesis to define established knowledge and pinpoint specific directions for future research. Enzyme-assisted conversion of aquaculture species and their processing by-products into antioxidant peptides is an expanding research frontier, reinforced by advances in other freshwater fish species, such as carp [8] and tilapia [7]. Yet, compared with carp and tilapia, comprehensive synthesis of knowledge on catfish-derived antioxidant peptides and protein hydrolysates, especially from a food science perspective, is currently limited.

The purpose of this review is to consolidate the findings of the past decade on catfish-derived protein hydrolysates and peptides, covering their production, characterization, and antioxidant efficacy. It emphasizes enzymatic hydrolysis strategies, structure–activity relationships, and applications in food systems, highlighting catfish as a sustainable source of natural antioxidants. A literature search was conducted using the Scopus database to identify primary research articles focusing on catfish-derived antioxidant peptides and hydrolysates. We searched the “Article title, Abstract, Keywords” fields using the keyword combination: “catfish” AND “antioxidant” AND (“peptide” OR “hydrolysate”). Primary inclusion criteria were limited to primary research articles published in English or with English abstracts between 2015 and 2025. From the initial 46 publications, following abstract screening, 22 studies were selected for detailed critical analysis. Review articles were excluded to prioritize original data. Relevant papers published prior to 2015 were cited, where appropriate, to provide necessary context regarding established knowledge, such as taxonomy, waste statistics, biochemical principles, and methodology. The subsequent discussion encompasses (i) the sustainability of catfish as a bioresource, (ii) peptide production methods, (iii) antioxidant efficacy and mechanisms, and (iv) key research gaps and future directions.

## 2. Catfish as a Sustainable Bioresource

### 2.1. Catfish Biodiversity and Global Aquaculture Production

The catfish group (order Siluriformes) is one of the most diverse orders of ray-finned fish. It contains more than 3000 species, making it one of the largest orders of teleosts [22,23]. The group has about 40 families distributed around the globe with the highest diversity in tropical South America, Africa, and Asia. Catfishes are a diverse group that fill critical ecological roles in aquatic food chains as predators, scavengers, and regulators of population dynamics [24,25]. They constitute an important food source for local communities and contribute to income generation and livelihood [26].

Catfishes represent a significant seafood resource due to their economic value, nutritional benefits, and suitability for high-efficiency aquaculture. With global production approaching 6.9 million metric tons [27], catfish provide a vital food resource, particularly in developing nations. Their production does not require sophisticated technology [25]. In 2023, global catfish production comprised 37 species with *Pangasius* (3.41 million metric tons) contributing 49.5% of the total production, followed by 1.84 million metric tons (26.6%) of airbreathing catfishes (mainly *Clarias* species), bagrid catfishes at 9.9% (0.68 million metric tons), and ictalurids (mostly channel catfish) at 8.8% (0.60 million metric tons). These four major catfish subgroups accounted for 95% of the total catfish production. The most cultured catfish species are striped catfish (*Pangasianodon hypophthalmus*) (locally known as Tra in Vietnam), torpedo-shaped catfish (*Clarias* spp.), yellow catfish (*Tachysurus sinensis*), channel catfish (*Ictalurus punctatus*), Pangas catfish (*Pangasius* spp.) and African catfish (*Clarias gariepinus*) (Table 1).

Vietnam’s *Pangasius* production reached 155,800 metric tons by March 2025. Its exports spanned key markets across Asia, South America, Africa, and North America. International demand for *Pangasius* remains robust, with 118 countries importing approximately 180,000 metric tons of frozen *Pangasius* in the first quarter of 2025, 93% of which was supplied by Vietnam [29].

### 2.2. Catfish By-Products as a Valorization Opportunity

Vietnam is considered a global leader in farming striped catfish (*P. hypophthalmus*) where about 900,000 metric tons of catfish by-products, including heads, viscera, trimmings, frames, skin, liver, and roes, are discharged annually [30,31]. In catfish processing, particularly for *P. hypophthalmus*, by-products can account for 60–65% of the total fish weight [18,32]. The *P. hypophthalmus* fillet accounts for 33–38% of the processed fish. Fish oil comprised over 15.3% of the fish weight, a significant component of the catfish (*P. hypophthalmus*) by-product waste [33]. Gelatin, which is an important raw material in various industries, such as pharmaceuticals and cosmetics, can be derived from the skin of *C. gariepinus* [34,35], *I. punctatus* [36], and *P. hypophthalmus* [37]. Gelatin has been extracted from the skin and bone of *P. hypophthalmus*, yielding 17.29% and 14.16% respectively [33].

### 2.3. Protein and Amino Acid Compositions

Catfish muscle is widely recognized as a high-quality protein source. Catfish muscle tissue typically contains 14–19% crude protein (wet weight basis) [38,39,40,41,42,43,44,45,46], although it can vary significantly based on farming systems and dietary conditions [38,39,40,42,47,48,49]. While commercial species like *C. gariepinus* [46,48,50,51] and *Pangasius* [39,45,52] often fall within this range, experimental conditions have yielded wider extremes [39,40,42,47,53]. For example, protein levels as low as 10.95% have been observed in muscle tissue of *Pangasius* reared in specific biofloc-probiotic systems [47]. On the other hand, a higher crude protein content (20.04%) was observed in juvenile channel catfish (*I. punctatus*) that were fed diets containing enzymatic fish paste [53].

The direct use of high-value fish fillets for industrial peptide extraction is economically impractical due to their high market value as human food [54]. By contrast, processing by-products of commercially important catfish species and muscle tissue from non-commercially important catfish species represent cost-effective alternatives. Non-fillet biomass is a promising raw material for extractable protein. For instance, *C. gariepinus* heads can be processed into fishmeal with a protein content of 48.42% (dry weight basis), while skin-derived collagen and gelatin possess 89.09% and 77.44% protein content, respectively [35]. Similarly, muscle of the invasive armored catfish (*P. disjunctivus*) is also a protein-rich raw material (19.8% on a wet weight basis) [55], comparable to that of commercially important catfish, making it suitable for possible antioxidant peptide extraction.

From *P. hypophthalmus* waste, pink slime meat, a processing waste mechanically recovered from the bone frames, can be refined into a dried protein concentrate containing 75.34% protein on an as-is basis, with residual moisture content of 8.82% [19]. Amino acid profiling (Table 2) shows that the pink slime protein concentrate contains substantial concentrations of leucine (63.5 mg/g dry sample) and valine (41.8 mg/g dry sample). These aliphatic amino acids are associated with antioxidant activity [55]. This, therefore, renders the protein concentrate a promising resource for valorization [19]. Beyond the recovered meat, the bone frames of *P. hypophthalmus* were also valorized for gelatin extraction. Unlike the myofibrillar profile of pink slime meat, the bone gelatin (Table 2) is dominated by glycine (157.1 mg/g) and proline (79.0 mg/g) [56]. The proline-enriched content of the bone gelatin is notable because it suggests that hydrolysates derived from these collagenous sources would exhibit antioxidant activity [57], while also possessing stability against gastrointestinal proteases due to proline’s cyclic amino ring impeding cleavage by enzymes such as pepsin and trypsin [58,59,60]. It should be noted that while comparisons can be drawn between studies sharing the same units, values expressed in different reporting metrics (i.e., mg/g protein versus mg/g dry sample) are not directly comparable. Therefore, Table 2 mainly serves to illustrate the relative abundance of amino acids and the inherent functional residue profiles across different catfish biomasses.

## 3. Production Strategies: Enzymatic Hydrolysis and Alternative Methods

### 3.1. Pre-Processing and Protein Isolation

Various anatomical samples of catfish, including muscle [15,62], skin [63,64], roes [65], viscera [13] and other fish by-products [21,66], have been used for hydrolysate production. Although the valorization of processing waste represents the main sustainability goal, muscle tissue has often been used in studies to establish optimal enzymatic kinetics and hydrolysis parameters [15,62]. Prior to enzymatic hydrolysis, both muscle and by-product samples typically undergo pre-processing steps, such as mincing, and homogenization. Given the high lipid content of catfish by-products (see Section 2.2), a common pre-processing step reported across studies involved defatting using organic solvents [63,65] and centrifugation [67]. In addition, heating and boiling of fish samples (typically at 85–100 °C for 10–20 min) were widely applied in these reported studies during the raw material pre-processing, in order to inactivate the endogenous fish enzymes [12,13,15,21,62,68]. Both the defatting and heating processes of fish samples facilitated the success of the subsequent enzymatic hydrolysis process. Following mechanical preparation (mincing, homogenization, and centrifugation), protein isolation was achieved through the pH-shift method [20], or acid precipitation [14,17]. For catfish skin samples, pre-processing with NaOH and acetic acid was performed to facilitate the extraction of gelatin and collagen proteins [11,63,64,69].

### 3.2. Enzymatic Hydrolysis

To prepare catfish protein hydrolysates, the isolated catfish proteins were next subjected to different protein hydrolytic processes. Table 3 summarizes the hydrolysis conditions yielding maximum antioxidant activity. Among the reported hydrolytic processes, the enzymatic approach remains the method of choice, which covers commercial proteases, by-product enzymes, and biological systems.

Alcalase was widely used for catfish protein hydrolysate production [12,14,15,20,21,55,62,68,73]. Other reported enzymes included bromelain [11,17,67,71]; Flavourzyme [14,66]; papain [13,17,67]; Neutrase [55,67]; and collagenase, trypsin, pepsin [13,63]. Some studies have also successfully used proteases extracted from catfish processing by-products, such as alkaline-like proteases from intestinal tissue [62] and peptidases from viscera [64,69]. These by-product-derived enzymes often matched or exceeded the effectiveness of commercial proteases in terms of hydrolysis rate, degree of hydrolysis (DH), and resulting antioxidant activity [62,69]. This highlights their dual role in valorizing waste streams and reducing the costs of enzymes. Furthermore, mixed enzyme treatments (combinations of collagenase with pepsin or trypsin) have been shown to outperform single enzyme hydrolysis in releasing antioxidant peptides [63].

Advanced hydrolysis designs have been explored, including a two-stage serial hydrolysis process using commercial Alcalase [65]. The two-stage serial hydrolysis was designed to avoid feedback inhibition by intermittently removing the soluble hydrolysate. This improves protein hydrolysate recovery and allows further digestion of compact protein fractions whose buried peptide bonds became more accessible after the first hydrolysis stage [65]. Furthermore, biological enzymatic approaches, including natural fermentation [16], and simulated gastrointestinal (GI) digestion models [64,70], have also been shown to release antioxidant peptides from catfish matrices through the action of microbial and digestive enzymes, respectively. Collectively, a comparative evaluation of these diverse strategies reveals two advancements over standard, single-enzyme commercial protocols. First, alternative approaches like by-product enzymes and mixed-enzyme systems often achieve superior antioxidant activity, with by-product enzymes offering the added advantage of lowering costs to drive a sustainable circular bioeconomy. Second, advanced processing designs, such as multi-stage hydrolysis, enhance targeted peptide release and improve overall hydrolysate recovery.

To produce a catfish protein hydrolysate with satisfying antioxidant potential, hydrolysis time and DH were frequently used as optimization parameters. Some studies used statistical experimental designs (e.g., 3^2^ factorial, Response Surface Methodology (RSM)) to identify optimal hydrolysis conditions that balance DH and antioxidant output [55,71]. Several studies reported a direct proportional relationship between DH/hydrolysis time and antioxidant potential [17,70]. For instance, one study investigating in vitro digestion of *I. punctatus* muscle reported that longer digestion time led to increased antioxidant capacity [70]. A similar trend was reported in a study preparing catfish muscle hydrolysate using papain. The study indicated that while hydrolysis activity increased over time, a hydrolysis duration of 30 min was sufficient to yield satisfying antioxidant activity, though the activity continued to rise slightly up to 90 min [17].

However, the relationship between DH and antioxidant activity also depends on the types of antioxidant assays used. Moreover, the inherent specificity of the protease, which determines which peptide bonds are cleaved, and therefore which peptide sequences are released, may be more influential than the DH itself in determining antioxidant efficacy [62,67]. For example, a 30% DH yielded the highest 2,2-diphenyl-1-picrylhydrazyl (DPPH) activity (with semi-purified intestinal protease) and the highest ABTS scavenging activity (with Alcalase). By contrast, a 20% DH yielded the highest ferric reducing antioxidant power (FRAP) for both enzymes [62]. Consequently, hydrolysis methods, sample type, and assay selection are interlinked factors in the optimization of DH and antioxidant efficacy.

### 3.3. Non-Enzymatic Hydrolysis Strategies

Besides the enzymatic approach, chemical and physical hydrolysis approaches were also reported. Chemical hydrolysis methods (using strong acid and base) were applied for the production of hydrolysate from catfish (*Pangasius* sp.) visceral proteins [13]. Sonication, a physical method, has been explored for the hydrolysis of collagen from catfish swim bladder tissue [72]. Unlike the direct correlation often observed in enzymatic hydrolysis [14,62,64,70], the study using sonication reported a non-linear relationship between processing time and antioxidant potential. Among the multiple tested durations (0–20 min), the protein hydrolysate P2 (15 min sonication) yielded the highest ABTS antioxidant activity [72].

## 4. Antioxidant Efficacy: Mechanisms, Structure-Activity Relationship (SAR), and Validation Models

### 4.1. Mechanisms and SAR

**Figure 1:**
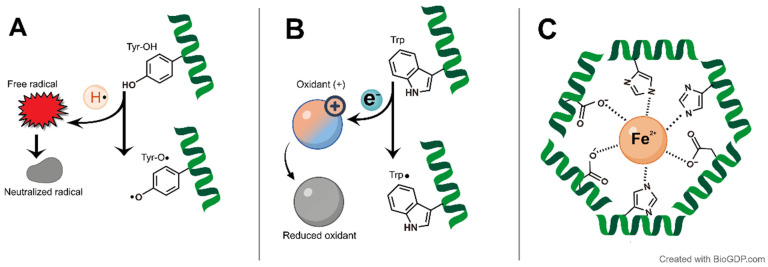
Schematic representation of the proposed primary antioxidant mechanisms of catfish-derived peptides. (**A**) Hydrogen Atom Transfer (HAT): A tyrosine residue donates a hydrogen atom (H•) to directly neutralize a free radical, resulting in the formation of a stable tyrosyl radical (Tyr-O•). (**B**) Electron Transfer (ET): A tryptophan residue transfers an electron (e^−^) to reduce an oxidant, leading to the formation of a tryptophyl radical (Trp•). (**C**) Metal Ion Chelation: Peptide fragments function as ligands to sequester pro-oxidant transition metals (Fe^2+^). The coordination cage is stabilized by imidazole nitrogen atoms (from Histidine) and negatively charged oxygen atoms (from Aspartate and Glutamate). In the schematic diagrams, green ribbons represent protein secondary structures (alpha-helices), while colored spheres and chemical structures illustrate the molecular interactions described. Created with BioGDP.com [77].

The primary antioxidant mechanisms reported for catfish-derived protein hydrolysates and peptides include hydrogen atom transfer (HAT) and electron transfer (ET) pathways [15,21,55] (Figure 1A,B). HAT-based mechanisms can be assessed using assays such as oxygen radical absorbance capacity (ORAC), which measure the ability of an antioxidant to neutralize peroxyl radicals (ROO·) by donating a hydrogen atom. In contrast, assays dominated by ET-based reactions, such as FRAP, evaluate the capacity of antioxidants to transfer an electron to an oxidant rather than directly interacting with radicals. Assays like DPPH and ABTS scavenging assays involve reaction pathways in which both HAT and ET mechanisms occur simultaneously, allowing them to capture mixed-mode antioxidant activities [74]. Most investigations used high-throughput chemical assays, such as DPPH, ABTS, and FRAP (Table 4). These in vitro assays offer certain strengths, as they are highly reproducible, rapid, and cost-effective platforms for initial peptide screening. However, their major limitation is the absence of true physiological relevance, as they cannot replicate bioavailability constraints, cellular phase partitioning, or endogenous antioxidant interactions of living systems [75,76]. Despite these limitations, the convenience of these chemical screens has led to over-dependence on them in the current catfish literature. Few studies have characterized the antioxidant activity of catfish-derived protein hydrolysates and peptides through the scavenging of physiologically relevant radicals such as hydroxyl (HO•) and peroxyl radicals [16,55], or validated antioxidant effects in in vivo models [73].

In the chemical assays that dominate the literature, the antioxidant potency of catfish-derived protein hydrolysates and peptides is often influenced by their structural characteristics, particularly molecular weight (MW). Studies often link the antioxidant efficacy of catfish peptides to their MW, with smaller peptides showing stronger antioxidant potency [7,8,78]. For example, the <1 kDa peptide fraction derived from Tra catfish (*P. hypophthalmus*) by-products exhibited superior radical scavenging and reducing power compared to their larger peptide fractions [21]. However, evidence from catfish hydrolysates suggests that the optimal peptide size depends largely on the specific antioxidant mechanism being tested. For instance, in silver catfish (*Pangasius* sp.) hydrolysates, while the smallest peptides (<3 kDa) were the most effective at scavenging radicals and reducing iron, larger peptides (5–10 kDa) were the best metal chelators [15] (Figure 1C). It is proposed that while the smaller peptides derive their efficacy from electron-donating hydrophobic residues, the larger peptides likely retain the structural features required for trapping metal ions that are lost during subsequent fractionation to smaller sizes [15]. This dependence on mechanism and structure is also exemplified in the findings from hydrolysates prepared from engraved catfish (*Nemapteryx caelata*) roe via two-stage serial hydrolysis. Lower MW peptides from the second stage (RH-2) showed better reducing power, whereas the higher MW fractions from the first stage (RH-1) had better radical scavenging activity [65]. This suggests that owing to extended hydrolysis, the smaller peptides of RH-2 likely lose the hydrophobic regions necessary to interact with radicals, whereas the larger peptides retain these important hydrophobic domains [65]. However, this size–activity relationship must also be considered in the context of physiological relevance. Although catfish-specific bioavailability data is unavailable, general peptide science suggests that smaller peptides (di- and tri-peptides) are generally preferred for intestinal absorption via the PepT1 transporter [79,80]. Thus, optimal antioxidant efficacy in vivo likely requires a balance, whereby peptides must be small enough for bioavailability but still retain specific hydrophobic sequences necessary for activity.

**Figure 2:**
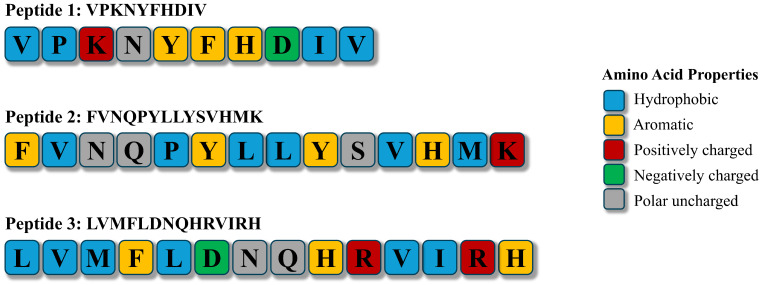
Amino acid sequences and properties of three antioxidant peptides identified from Patin catfish (*Pangasianodon hypophthalmus*) myofibrillar protein hydrolysate [14]. Residues are color-coded according to physicochemical properties: hydrophobic (blue), aromatic (yellow), positively charged (red), negatively charged (green), and polar uncharged (gray).

Hydrophobic amino acids (e.g., Ala, Val, Leu, Ile, Phe, Pro, Met) are frequently associated with antioxidant activity, potentially facilitating interaction with lipid-rich membranes. Meanwhile, aromatic residues (e.g., His, Trp, Tyr, Phe) contribute to radical scavenging and metal chelation [14,55,78] (Figure 1). This SAR was validated by Najafian and Babji [14], who identified three antioxidant peptides (VPKNYFHDIV, LVMFLDNQHRVIRH, and FVNQPYLLYSVHMK) from Patin catfish. These peptides have potent DPPH scavenging activity (IC_50_ 0.268–0.443 mg/mL) and lipid oxidation inhibitory activity (thiobarbituric acid reactive substances [TBARSs]: 1.75–2.00 mg MDA/kg). Their efficacy supports the idea that the specific arrangement of hydrophobic residues (e.g., Phe, Val, Leu) at the N-terminus and often basic residues (e.g., Lys) at the C-terminus, combined with aromatic residues (e.g., Tyr, His) within the sequences, allow these peptides to effectively end radical chain reactions and suppress lipid oxidation [14]. The distribution of hydrophobic and aromatic residues within these peptide sequences, which is critical for their antioxidant activity, is depicted in Figure 2. However, an exception exists, such as the pepsin hydrolysates from catfish (*Pangasius* sp.) viscera, which exhibited superior scavenging activity despite lower hydrophobicity [13]. This suggests that steric positioning may override hydrophobicity; specific peptide sequences exposed by pepsin likely allow for more efficient electron transfer despite a lower abundance of hydrophobic and aromatic residues [13].

A review of the available catfish peptide literature revealed that sequence-specific insights are rare. To date, only Najafian and Babji [14] have identified and experimentally validated three antioxidant peptide sequences (e.g., VPKNYFHDIV) from Patin catfish (*P. hypophthalmus*). Other studies have reported approaches that yielded incomplete information. For example, in recent studies on armored catfish (*P. disjunctivus*) [55] and fermented hybrid catfish (*C. macrocephalus* × *C. gariepinus*) [16], researchers used peptidomic profiling to identify putative antioxidant peptides from three active hydrolysates (56 sequences) [55] and a peptide fraction (85 sequences) [16]. Although the profiled peptides reported in the two latter studies [16,55] are rich in hydrophobic and aromatic amino acids, which are characteristics correlated with antioxidant activity, they were not synthesized and tested individually to confirm their antioxidant activity. In contrast, some research [13,20,62,71] has reported only amino acid compositions of catfish protein hydrolysates, without sequence information. While these findings may suggest links between amino acid types and antioxidant activity, they cannot reveal how sequence-specific factors, such as order, length, or motifs, influence antioxidant mechanisms [81]. This limitation is critical because antioxidant efficacy is not merely additive. Amino acid composition profiles treat amino acids as interchangeable contributors. However, antioxidant activity often relies on interactions between residues. Consequently, amino acid composition data inherently loses this structural context [81]. Therefore, a precise SAR cannot be established until the complete sequences of catfish-derived antioxidant peptides are identified through proteomic profiling, followed by the direct validation of their antioxidant efficacy.

### 4.2. Validation in Chemical, Biological, and Food Systems

The antioxidant efficacy of catfish protein hydrolysates and peptides has been validated largely through in vitro chemical assays (Table 4). It should be noted that the majority of antioxidant data presented in Table 4 are derived from complex, unfractionated protein hydrolysates rather than purified individual peptides. Consequently, the reported bioactivities reflect the collective performance of peptide mixtures, where the specific molecular contributions or synergistic interactions in the hydrolysate are largely uncharacterized. Muscle protein hydrolysates from the armored catfish (*Pterygoplichthys disjunctivus*) exhibited potent antioxidant capacities. Specifically, the Neutrase hydrolysate of the species achieved an ABTS scavenging value of 174.68 µmol TE/g fish and a FRAP value of 7.59 mg AAE/g fish [55]. The armored catfish is an invasive species which accounts for up to 80% of the total catch in affected reservoirs. Thus, using the species as a source of bioactive ingredients, particularly natural antioxidants, serves not only as a strategy for waste reduction but also as an economically sustainable approach to tackle ecological contamination [55].

Beyond invasive species, catfish hydrolysates have consistently demonstrated high efficacy in standard antioxidant chemical assays. Myofibrillar protein hydrolysates from Patin catfish (*P. hypophthalmus*) exhibited potent scavenging activity against DPPH and superoxide radicals, with antioxidant efficacy increasing alongside a higher degree of hydrolysis (DH) and improved peptide solubility [14]. Similarly, studies on protein hydrolysates derived from bighead carp and rainbow trout showed that higher DH enhanced functional characteristics, particularly protein solubility and DPPH scavenging activity [82,83].

Research comparing enzyme sources indicated that a semi-purified intestinal protease applied on Chihuil sea catfish (*Bagre panamensis*) muscle yielded a hydrolysate with higher DPPH radical scavenging activity (118.8 µmol TE/mg) than a hydrolysate produced by commercial Alcalase (103.0 µmol TE/mg) [62]. Similarly, Mekong giant catfish (*Pangasianodon gigas*) skin gelatin hydrolyzed by visceral peptidase exhibited an ABTS scavenging activity of 176.24 µmol TE/g protein, performing more effectively than bovine trypsin [69]. Together, these findings substantiate the potential of by-product enzymes for producing high-value antioxidants from catfish matrices and likely other fish.

Peptide stability under GI conditions is critical for nutraceutical applications. Studies on skin gelatin hydrolysates from Mekong giant catfish (*P. gigas*) subjected to pepsin and pancreatin digestion revealed that antioxidant activity was generally maintained or even enhanced [64,69]. However, it is vital to explicitly differentiate between bioaccessibility (digestive stability) and systemic bioavailability (intestinal absorption). While these in vitro GI digestion models confirm that the peptide mixtures resist complete enzymatic degradation, they do not provide any information regarding epithelial transport or plasma stability. Notably, trypsin-derived skin gelatin hydrolysates exhibited an over 100-fold increase in ABTS radical scavenging activity post-digestion [64], while DPPH scavenging activity also increased significantly [69]. This suggests that the sequences of putative antioxidant peptides are either resistant to GI proteases or are cleaved into smaller, more potent fragments, supporting their viability as oral nutraceuticals. This enhancement of activity does not contradict the earlier in vitro observation that extensive hydrolysis can diminish specific antioxidant capacities, such as radical scavenging. As established in Section 4.1, the relationship between peptide size and antioxidant efficacy is highly dependent on the specific type of antioxidant activity being evaluated. Specifically, while in vitro over-hydrolysis using broad-spectrum commercial proteases can degrade the larger hydrophobic domains required for certain radical-trapping interactions, simulated GI digestion uses highly specific physiological proteases. For example, pepsin preferentially cleaves peptide bonds adjacent to aromatic and hydrophobic amino acids [84]. Instead of destroying the peptide’s overall antioxidant potential, this targeted cleavage strategically exposes terminal aromatic and hydrophobic residues. Consequently, the resulting small fragments may gain highly potent electron-donating or metal-chelating capacity, distinguishing simulated GI digestion from generic in vitro over-hydrolysis [57].

Furthermore, processing conditions and inherent substrate sequences also appear to influence this stability. For instance, the GI digestate of heat-treated channel catfish (*I. punctatus*) muscle exhibited higher antioxidant activity when compared to the digestate of raw non-pre-heat-treated meat [70]. Conversely, if a substrate has already been extensively pre-hydrolyzed during processing (such as fermentation), subjecting these already-short peptides to simulated GI assay may lead to over-hydrolysis, further degrading the peptides and destroying their functional antioxidant domains. For example, the radical scavenging activity of a peptide fraction prepared from fermented hybrid catfish (*Clarias macrocephalus* × *C. gariepinus*) was reduced following simulated duodenal digestion [16]. Taken together, these findings suggest that peptide stability is enzyme-, substrate-, and processing-dependent.

In vivo validation of the antioxidant efficacy of catfish-derived protein hydrolysates and peptides is limited. To date, only Kim et al. [73] have reported such evidence in a murine model. In a study targeting obesity, mice fed a 45% kcal high-fat diet supplemented with yellow catfish (*T. sinensis*) protein hydrolysate showed significantly suppressed body weight gain and white adipose tissue accumulation compared to controls [73]. Furthermore, the hydrolysate treatment restored hepatic glutathione (GSH) content and antioxidant enzyme activities (SOD and CAT) which were suppressed by the high-fat diet, besides reducing liver lipid peroxidation, thus demonstrating protective antioxidant effects in vivo [73]. While Kim et al. [73] demonstrated that these antioxidant defenses were restored, the exact cellular mechanisms for catfish peptides remain unknown. However, research on other food peptides shows that such biological antioxidant activity is mostly driven by the modulation of mitochondrial ROS and the activation of the Keap1/Nrf2 signaling pathway [85]. By promoting Nrf2 nuclear translocation, peptides upregulate the synthesis of endogenous antioxidant defenses, including SOD, CAT, and GSH observed in the murine model. This biological response provides a much stronger protection than simply scavenging radicals directly [86]. Exploring these cellular pathways is the logical next step for catfish peptide research.

The stability of antioxidant protein hydrolysates and peptides under food processing conditions is an important consideration for application. For instance, it was demonstrated that the DPPH scavenging activity of *P. gigas* hydrolysate was resilient to prolonged heating at 100 °C [69]. Although the scavenging activity initially dropped, it recovered and exceeded the original level by 180–240 min. Furthermore, the study found that DPPH scavenging activity of the hydrolysate remained stable across a broad pH range (1–11) [69]. Similarly, a peptide fraction from fermented hybrid catfish (*Clarias macrocephalus* × *C. gariepinus*) showed increasing DPPH scavenging activity with rising temperature (60–100 °C) and greater stability in acidic versus alkaline conditions [16].

Incorporation of catfish hydrolysates into food matrices serves the dual purpose of functional fortification and shelf-life extension. In shortfin scad emulsion sausages, the addition of *Pangasius* sp. protein hydrolysate (1–3%) significantly inhibited lipid oxidation, as evidenced by consistently lower peroxide value (PV) and TBARS compared to the control during storage [12]. Hamzah et al. [12] also reported that the hydrolysate improved the textural properties of the emulsion sausage. Similarly, Halmi et al. [68] claimed that the incorporation of 3% catfish protein hydrolysate helped to maintain oxidative stability in shortfin scad and surimi emulsion sausages over 12 days of storage. However, a critical examination of their study design shows that the hydrolysate was added to all formulations tested, which differed in fish mince-to-surimi ratio. The formulation containing 100% surimi, which had the lowest inherent fat content, exhibited the lowest PV and TBARS values. Thus, the observed oxidative stability cannot be attributed to the protection by the hydrolysate. This highlights the need for well-controlled designs that can isolate the additive effect of a bioactive hydrolysate from other formulation variables in future food application studies.

**Figure 3:**
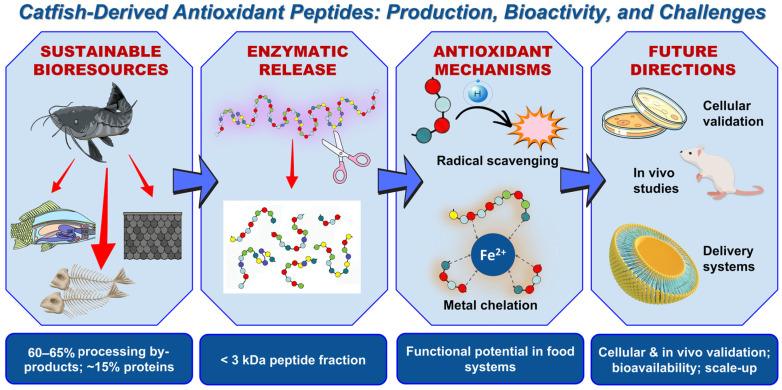
Schematic summary of the valorization pipeline for catfish-derived antioxidant peptides. The figure illustrates the conversion of sustainable processing by-products into low-molecular-weight peptides via enzymatic release, their core antioxidant mechanisms, and their functional potential in food systems. Key future directions required to validate their physiological efficacy and enable industrial application are also highlighted. The workflow is divided into four blue octagonal panels representing sustainable bioresources, enzymatic release, antioxidant mechanisms, and future directions (from left to right). Blue arrows indicate process progression. Icons depict specific elements: fish and frames (bioresources), scissors and peptide chains (enzymatic hydrolysis), radicals and Fe^2+^ (mechanisms), and mouse and delivery systems (validation and application). Bottom blue boxes summarize key parameters for each stage.

Overall, the successful conversion of catfish by-products into functional food ingredients relies on an integrated pipeline—from targeted enzymatic hydrolysis to a mechanistic understanding of the resulting peptides. A comprehensive schematic summary of this valorization process, highlighting the progression from sustainable bioresources to core antioxidant mechanisms and future application directions, is presented in Figure 3.

## 5. Challenges and Future Directions for Food Application

Despite advances over the past decade in characterizing catfish-derived antioxidant peptides and protein hydrolysates, as discussed above, a number of research gaps remain that limit their application as natural antioxidants in food systems. Addressing these gaps is essential to unlocking the full potential of catfish and their by-products as a sustainable source of food-grade antioxidants.

(i) **Limited biological validation**

Most studies relied primarily on in vitro chemical assays to evaluate the antioxidant activity of catfish-derived protein hydrolysates and peptides; but none employed cellular models to measure intracellular antioxidant activity. Only Kim et al. [73] have reported in vivo evidence, demonstrating that a yellow catfish (*T. sinensis*) hydrolysate enhanced antioxidant responses in mice fed a high-fat diet. This finding suggests the physiological efficacy of catfish-derived peptides in functional food applications. It warrants further cellular studies to elucidate the antioxidant mechanisms and additional in vivo trials to validate the nutritional benefits.

Cellular and in vivo studies are critical to confirm the physiological relevance of antioxidant peptides and protein hydrolysates derived from catfish, as chemical assays cannot fully replicate the complex biological environments of cells and tissues [75,76]. Although in vitro and animal models have demonstrated their antioxidant efficacy, comprehensive cytotoxicity validation of catfish-derived protein hydrolysates and peptides using fibroblasts, hepatocytes, or intestinal epithelial cells is needed. While a previous study concluded that collagen hydrolysate from catfish skin was safe for human consumption, it relied solely on a red blood cell coagulation assay [63], which is insufficient for broad safety claims. Thus, future research should prioritize cell-based assays, such as measurements of cellular antioxidant activity and ROS inhibition in HepG2 cells [87,88], alongside investigations into mitochondrial ROS regulation and Keap1/Nrf2 pathway activation, to evaluate oxidative stress protection by catfish-derived antioxidant peptides and protein hydrolysates. Such experiments can expedite the development of a foundation of biological evidence to substantiate the application of catfish antioxidant peptides and protein hydrolysates as functional food ingredients. Moreover, these results can inform subsequent in vivo trials to validate efficacy in dietary applications.

(ii) **Incomplete peptide characterization**

Investigations into the SAR of catfish-derived antioxidant peptides are limited. This knowledge gap limits the rational design of targeted antioxidant peptides for specific food systems. To address this gap, future research should prioritize peptide sequence identification using Liquid Chromatography-Tandem Mass Spectrometry [89]. However, the identification of peptides from non-model organisms like catfish is challenging [90]. Traditional database-dependent workflows often yield low identification rates owing to the poorly annotated proteomes of these species when compared to model organisms [90,91]. De novo sequencing offers an alternative by deriving sequences directly from fragmentation spectra [92]. Nonetheless, it is computationally intensive and prone to errors, particularly in differentiating isobaric amino acids such as leucine and isoleucine [93]. Overcoming these challenges likely requires integrating de novo algorithms with custom, species-specific transcriptomic databases to ensure high-confidence sequence identifications [91,94]. When coupled with subsequent functional validation, this will advance SAR knowledge, which in turn will drive the development of optimized antioxidant peptides with predictable efficacy for use in food systems.

(iii) **Stability under diverse food processing conditions**

For catfish-derived peptides to be applied as food ingredients, they must retain antioxidant activity under relevant processing and storage conditions, including thermal treatment, pH variation, freeze–thaw cycles, and high salt conditions [95,96]. At present, data on such practical stability remains limited (see Section 4.2) [12,16,68,69]. In contrast, other studies focused only on in vitro antioxidant activities of catfish-derived peptides and protein hydrolysates. The authors did not test stability under conditions relevant to food production, which limits confidence in their practical applications.

Future research should systematically investigate stability across diverse processing conditions, such as high-temperature cooking and acidic food matrices. Long-term storage studies (e.g., 6 months at 25 °C, 4 °C, or −20 °C) are also needed to ensure peptides maintain antioxidant activity in real-world food applications. Regarding food processing and applications as food ingredients, future investigations into the interactions of catfish-derived peptides and protein hydrolysates with food components (e.g., lipids, carbohydrates, metals) that may enhance or inhibit their antioxidant activity are also warranted.

(iv) **Sensory and consumer acceptance**

Sensory and textural properties are crucial for consumer acceptance when incorporating bioactive peptides into foods [97,98]. However, systematic sensory profiling of catfish-derived antioxidant hydrolysates is limited. Existing studies have focused primarily on textural properties or oxidation inhibition rather than detailed flavor and aroma profiling (see Section 4.2) [12,68]. Halmi et al. [68] claimed that incorporating silver catfish hydrolysate (3%) into fish sausages resulted in low bitterness, although this was inferred only from general taste preference scores from an untrained panel. Ayat and Shakir [63] reported “bitterness” in catfish skin collagen hydrolysates, although the specific methodology for this determination was not provided. Thus, the lack of methodological rigor in these studies is a critical gap in the current literature.

Bitterness and fishy off-flavors are commonly associated with fish protein hydrolysates [9]. The same hydrophobic amino acids and low-MW peptides that confer strong antioxidant activity also taste bitter. When proteins undergo extensive hydrolysis, low-MW peptides are released and their hydrophobic residues are exposed, allowing them to interact with bitter taste receptors on the tongue [99,100]. This bitterness restricts the application of catfish-derived hydrolysates and peptides in diverse food products.

Glycation of protein hydrolysates derived from channel catfish (*I. punctatus*) viscera with glucosamine reduced bitterness and masked fishy odors [101]. The findings indicate that Maillard-based flavor modification is a promising strategy to improve sensory profiles. Future research should use trained panels to verify whether flavor-masking techniques, such as Maillard-based glycation or encapsulation, can deliver palatable ingredients without sacrificing their antioxidant capacity.

(v) **Bioavailability and delivery systems**

Bioavailability data for catfish-derived protein hydrolysates and peptides are scarce. To date, only Kim et al. [73] have reported in vivo antioxidant effects in mice, whereas Ketnawa et al. [64], Zhang et al. [70], and Chaijan et al. [16] focused on in vitro GI digestion stability. Importantly, there is an absence of intestinal epithelial transport studies evaluating catfish-derived antioxidant peptides in the current literature. To bridge the translational gap between in vitro digestive stability and physiological efficacy, future research must incorporate transepithelial transport models, such as Caco-2 cell monolayers or ex vivo Ussing chamber assays. These models are necessary to determine whether these peptides can successfully navigate PepT1-mediated transport or paracellular pathways to enter the systemic circulation intact. Furthermore, no research has explored the use of delivery systems, such as encapsulation, to protect catfish-derived antioxidant peptides during digestion and enhance their bioavailability. Encapsulation techniques, such as those using liposomes and nanogels, could improve peptide stability and bioactivity in functional foods [102]. Future research should investigate the GI fate of catfish-derived protein hydrolysates and peptides using in vivo and in vitro models. Researchers should also develop encapsulation techniques to improve the stability, bioactivity, and nutritional benefits of these protein hydrolysates and peptides in functional foods.

(vi) **Reproducible enzyme sourcing and scalable production**

One barrier to translating catfish-derived antioxidant peptides and protein hydrolysates into commercial food ingredients is the lack of reproducible enzyme sourcing and the use of unvalidated scalable production technologies. Most studies rely on common proteases but omit details that ensure results can be reproducible. For example, catfish collagenase was demonstrated to outperform pepsin and trypsin in producing catfish skin collagen hydrolysates with superior antioxidant activity [63]. However, reproducing the results using catfish collagenase would be challenging since the study provided no details on how this collagenase was prepared, purified, or standardized. Likewise, Baehaki et al. [11] and Nurdiani et al. [71] used bromelain for hydrolyzing catfish by-products, but did not report the enzyme’s specific activity, and in the case of Baehaki et al. [11], even the supplier was not specified. Commercial bromelain preparations differ in proteolytic activity depending on source and purity; hence, reporting enzyme activity is essential for reproducibility. The same concern applies to Najafian and Babji [14], who used papain for Patin catfish (*P. hypophthalmus*) hydrolysate preparation without specifying its activity.

On the other hand, scalable processing is also underexplored. Most research has used small lab batches, and only Hassan et al. [13] tested an industrial method: spray drying *Pangasius* sp. visceral hydrolysate into stable powder. Notwithstanding, this work did not address industrial priorities like energy efficiency of spray drying or batch-to-batch consistency in hydrolysate quality, which are key for food manufacturers. In contrast, an integrated strategy combining enzymatic hydrolysis and glycation in a single thermal step was developed to simultaneously inactivate Flavourzyme and initiate the Maillard reaction [101]. This represents a process-efficient, food-compatible strategy that can be scaled for industrial production of catfish viscera hydrolysates. Furthermore, even with scalable processing, the use of undefined hydrolysate mixtures presents a challenge for reproducibility and standardization. Unlike purified bioactive compounds, the efficacy of a protein hydrolysate is inherently sensitive to natural variations in raw catfish by-products (e.g., seasonal or dietary factors) and minor fluctuations in hydrolysis conditions. Without identifying and quantifying specific peptides to ensure quality control, achieving consistent batch-to-batch antioxidant potency would be a key barrier to the industrial commercialization and regulatory approval of these functional ingredients.

Future research should standardize enzyme reporting (activity, supplier, batch) to enable reproducibility and explore industrial-scale technologies, e.g., spray drying, continuous hydrolysis reactors, for catfish hydrolysate production. In addition, cost–benefit analyses should be conducted to confirm that catfish antioxidant peptides and protein hydrolysates can compete with common synthetic antioxidants in food markets.

## 6. Conclusions

Catfish and their processing by-products are a promising and sustainable source of natural antioxidant peptides. Current research supports their efficacy in chemical antioxidant assays, driven by low MW fractions and hydrolysates enriched in hydrophobic and aromatic amino acids. Regarding production, enzymatic hydrolysis using commercial Alcalase currently is the most promising strategy for releasing potent antioxidant peptides. Furthermore, the application of by-product-derived proteases represents a highly viable, cost-effective alternative that simultaneously advances a circular bioeconomy. At present, the field is still constrained by a heavy dependence on in vitro models, with minimal structural characterization apart from amino acid profiling, and limited validation in biologically relevant models. The most critical gaps that must be addressed include a complete absence of cellular antioxidant validation, the presence of inherent bitterness restricting food application, and a lack of standardized enzyme reporting that limits protocol reproducibility. Only limited in vivo data confirm the physiological antioxidant effects of the catfish protein hydrolysate, and cellular data are absent. Moreover, the use of catfish-derived protein hydrolysates in food matrices is hindered by sensory issues, inconsistent evidence for oxidative protection independent of formulation effects, and a lack of processing/storage stability data. To advance the field, future research must prioritize specific, actionable steps: (i) proteomic identification of antioxidant peptide sequences to establish the SAR and enable rational peptide design; (ii) elucidation of bioavailability and cellular antioxidant mechanisms using established cell lines; (iii) development of flavor-masking and encapsulation strategies to ensure consumer acceptance; and (iv) scalable, standardized production protocols (such as continuous hydrolysis or spray drying) supported by rigorous cost–benefit analyses to ensure these peptides can compete commercially with synthetic alternatives. Addressing these challenges will position catfish-derived antioxidant protein hydrolysates and peptides as viable, natural alternatives in the functional food and nutraceutical industries.

## Figures and Tables

**Table 1:** Global production (thousand tons, live weight) of major cultured catfish species.

Common Name	Scientific Name	Synonym	Year					
			2018	2019	2020	2021	2022	2023
Striped catfish, Tra, Patin	*Pangasianodon hypophthalmus*	*Pangasius hypophthalmus*, *Pangasius sutchi*	2360.73	2687.96	2580.48	2570.23	2850.38	2951.40
Torpedo-shaped catfish, Indonesian catfish	*Clarias* spp.	-	1132.08	1211.98	1120.15	1167.07	1241.36	1292.09
Yellow catfish, bagrid catfish	*Tachysurus sinensis*	*Tachysurus fulvidraco*	509.61	536.96	565.48	587.82	599.80	622.65
Channel catfish	*Ictalurus punctatus*	-	391.94	458.04	454.02	513.55	567.93	603.99
Pangas catfishes NEI	*Pangasius* spp.	-	449.11	468.97	443.99	436.15	449.45	442.49
African catfish	*Clarias gariepinus*	-	362.82	367.53	373.40	400.22	393.56	422.92

Data source: FAO [28].

**Table 2:** Amino acid composition of catfish muscle, by-products, and gelatin.

	Expressed as mg/g Protein			Expressed as mg/g Dry Sample	
Amino Acids	*Clarias gariepinus* Meat [61]	*Pangasianodon hypophthalmus* By-Product Hydrolysate [31]	*Pangasianodon hypophthalmus* Side-Stream Hydrolysate (Range) [20]	*Pangasianodon hypophthalmus* Pink Slime Protein Concentrate [19]	*Pangasianodon hypophthalmus* Bone Gelatin [56]
**Hydrophobic**					
Alanine	68.1	39.9	65–66	46.2	65.6
Glycine	52.3	40.9	33–34	28.9	157.1
Isoleucine	49.4	3.6	39–42	44.6	11.4
Leucine	87.2	7.4	85–88	63.5	22.6
Methionine	29.1	14.4	28–32	NA	NA
Phenylalanine	39.1	3.6	29–31	32.4	13.0
Proline	35.4	18.8	34–40	NA	79.0
Tryptophan	10.9	NA	NA	0.9	NA
Valine	52.3	12.5	43–48	41.8	18.8
**Total hydrophobic**	**423.8**	**141.1**	**356–381**	**258.3**	**367.5**
**Charged/Polar**					
Arginine	59.6	30.2	59–66	51.7	65.6
Aspartic acid	98.7	42.9	107–109	80.9	39.0
Cysteine	7.5	11.0	6–7	NA	NA
Cystine	NA	NA	6–7	NA	NA
Glutamic acid	159.0	38.4	192–204	123.3	71.9
Histidine	21.4	12.0	22–25	19.3	4.7
Hydroxyproline	NA	26.5	NA	NA	NA
Lysine	102.6	15.6	96–104	71.2	38.7
Serine	37.8	5.0	45–48	30.7	28.6
Threonine	50.7	12.5	44–45	35.8	22.2
Tyrosine	33.1	36.2	26–28	NA	4.4

NA, data not reported in cited study. Values for *P. hypophthalmus* pink slime protein concentrate were converted from % [19]. Suwondo et al. [19] reported Tyrosine and Phenylalanine as a combined value (3.18%), which contradicted the individual Phenylalanine value reported (3.24%); therefore, the specific Tyrosine content could not be determined. Suwondo et al. [19] reported Methionine and Cystine as a combined value (2.73%); therefore, specific Methionine content could not be determined. Values for *P. hypophthalmus* side-stream hydrolysate represent the range (min–max) across dark muscle, head and backbone blend, and abdominal cut-offs [20]. Values for *C. gariepinus* meat represent the mean of Black and Gray catfish meat [61]. Standard errors/deviations have been omitted for clarity. Total Hydrophobic is the sum of Alanine, Glycine, Isoleucine, Leucine, Methionine, Phenylalanine, Proline, Tryptophan, and Valine.

**Table 3:** Pre-processing and hydrolysis conditions for the production of catfish-derived antioxidant hydrolysates.

Catfish Species/Source	Tissue/By-Product Part	Pre-Treatment Steps	Hydrolysis Conditions for Antioxidant Activity	Reference
**I. Commercial Enzyme: Alcalase**				
*Nemapteryx caelata*	Roe	Homogenization; defatting with isopropanol	Two-stage serial hydrolysis using Alcalase: E/S 0.5% (*w*/*w*), pH 8, 50 °C, 2 h per stage	[65]
*Pangasianodon hypophthalmus*	Dark muscle (as protein isolate)	pH-shift method (solubilization at pH 12; precipitation at pH 5.5)	Alcalase: E/S 3% (*v*/*w*), pH 7.5, 50 °C, 3 h	[20]
	By-product (head, bone, fin, tail)	Heat inactivation (95 °C, 10 min)	Alcalase: E/S 30 U/g protein, pH 7.5, 55 °C, 5 h	[21]
*Pangasius* sp.	Muscle	Heat inactivation (85 °C, 20 min)	Alcalase: E/S 2.26% *, pH 7.89, 50 °C, 3 h	[15]
	Muscle	Heat inactivation (85 °C, 20 min)	Alcalase: E/S not reported, pH 7.9, 50 °C, 84 min	[12,68]
**II. Other Commercial Enzymes**				
*Clarias* sp.	Muscle (as protein isolate)	Alkali solubilization (pH 11, 4 °C); acid precipitation (pH 5.5, 4 °C); centrifugation	Papain: E/S 1% (*w*/*w*), pH 6, 37 °C, 30 min	[17]
*Ictalurus punctatus*	Muscle	Steaming (to core 70 °C)	Simulated GI digestion (INFOGEST method) on steamed muscle	[70]
*P. gigas*	Skin gelatin	NaOH (0.2 M, 2 h, 4 °C); acetic acid (0.05 M, 3 h, RT); water extraction (1:10 *w*/*v*, 45 °C, 12 h)	Izyme AL^®^: E/S 8 U/g protein, pH 8, 55 °C, 2 h	[64]
*Pangasius* sp.	Muscle (trimming by-product)	Skin and fat were removed prior to homogenization.	Bromelain optimized by RSM: E/S 0.04% *, pH 7, 55 °C, 2.8 h	[71]
	Visceral protein	Heat inactivation (100 °C, 10 min)	Pepsin: E/S 1% (*w*/*w*), pH 2, 37.5 °C, 5 h	[13]
*P. hypophthalmus*	Bone gelatin	Citric acid demineralization; hot water extraction (75 °C, 5 h)	Flavourzyme: E/S 6% (*v*/*v*), pH 7, 50 °C, 3 h	[66]
	By-product (shredded meat)	Heat treatment (100 °C, 10 min); defatting by centrifugation (10,000× *g*, 5 min)	Papain: E/S 2.76 mg/g, pH 7.5, 55 °C, 3 h	[67]
	Muscle (myofibrillar protein isolate)	Alkali solubilization (pH 11, 4 °C); isoelectric precipitation (pH 5.5, 4 °C)	Papain: E/S 1% (*w*/*w*), pH 7, 50 °C, 2 h	[14]
*Pterygoplichthys disjunctivus*	Fillet (muscle)	Minced in 0.1 M Bis-Tris propane buffer; pH adjusted	Neutrase HT^®^ L200: E/S 0.2% (*w*/*v*), pH 7.5, 50 °C, 2 h	[55]
Catfish (scientific name not specified)	Skin collagen	NaOH (0.1 M, 1:8 *w*/*v*); defatting with butyl alcohol (10%, 1:10 *w*/*v*); acetic acid extraction (0.5 M, 1% pepsin, 72 h)	Collagenase + Trypsin: E/S 1% *, pH 7.8, 37 °C, 90 min	[63]
**III. By-product Enzymes**				
*Bagre panamensis*	Muscle	Heat inactivation (90 °C, 15 min)	Semi-purified intestinal protease: E/S 0.188 U/g, pH 9, 40 °C, 30% DH	[62]
*P. gigas*	Skin gelatin	NaOH (0.2 M, 2 h, 4 °C); acetic acid (0.05 M, 3 h, RT); water extraction (1:10 *w*/*v*, 45 °C, 12 h)	Visceral peptidase: E/S 10 U/g protein, pH 8, 70 °C, 2 h	[69]
**IV. Non-Enzymatic Method**				
*Pangasius* sp.	Swim bladder (collagen)	NaOH (0.05 M, 1 h, 4 °C); citric acid (0.2%, 6 h, 4 °C); water extraction (1:1 *w*/*v*, 40 °C, 6 h)	Sonication: 20 kHz, 50% amplitude, 15 min, 10 °C	[72]

Abbreviations: DH, degree of hydrolysis; E/S, enzyme/substrate ratio; GI, gastrointestinal; RSM, response surface methodology; RT, room temperature. * Unit basis for E/S concentration not specified in original study.

**Table 4:** Comparative antioxidant properties of catfish protein hydrolysates and peptides across chemical, gastrointestinal digestion, in vivo, and food application models.

Hydrolysate/Peptide Source	Catfish Species	Antioxidant Activity/Parameter	Reference
**Chemical Assays—Muscle and Myofibrillar Proteins**			
Intestinal protease-hydrolyzed muscle (30% DH)	*Bagre panamensis*	DPPH: 118.8 μmol TE/mg ABTS: EC_50_ 1.5 mg/mL	[62]
Bromelain-hydrolyzed protein isolate	*Clarias* sp.	At 0.1 mg/mL: DPPH: 49.52% (13.07 mg/L AAE) Reducing power: A_700_ = 0.44	[17]
Papain-hydrolyzed protein isolate		At 0.1 mg/mL: DPPH: 59.58% (15.83 mg/L AAE) Reducing power: A_700_ = 0.80	
Peptide fraction	*Clarias macrocephalus* × *C. gariepinus*	DPPH: IC_50_ 0.23 mg/mL ABTS: IC_50_ 0.37 mg/mL HO•: IC_50_ 0.54 mg/mL H_2_O_2_: IC_50_ 0.35 mg/mL ^1^O_2_: IC_50_ 0.32 mg/mL Ferrous chelating: IC_50_ 3.21 mg/mL	[16]
Peptides identified from papain-hydrolyzed myofibrillar protein	*Pangasianodon hypophthalmus*	**VPKNYFHDIV:** DPPH: IC_50_ 0.354 mg/mL; TBARS: 1.92 mg MDA/kg **FVNQPYLLYSVHMK** DPPH: IC_50_ 0.268 mg/mL; TBARS: 2.00 mg MDA/kg **LVMFLDNQHRVIRH** DPPH: IC_50_ 0.443 mg/mL; TBARS: 1.75 mg MDA/kg	[14]
Hydrolyzed shredded meat (Enzymes: Neutrase, Papain, Bromelain)		DPPH (2 mg/L): Neutrase: 71.19% Papain: 70.60% Bromelain: No significant increase vs. control	[67]
Alcalase-hydrolyzed muscle (<3 kDa fraction)	*Pangasius* sp.	At 10 mg/mL: DPPH: 55.12% Ferrous chelating: 6.03%	[15]
Neutrase HT^®^ L200-hydrolyzed fillet	*Pterygoplichthys disjunctivus*	ABTS: 174.68 µmol TE/g fish FRAP: 7.59 mg AAE/g fish Peroxyl: 51.43 µmol TE/g fish	[55]
Neutrase PAL^®^ 660-hydrolyzed fillet		ABTS: 148.14 µmol TE/g FRAP: 5.82 mg AAE/g Peroxyl: 55.60 µmol TE/g	
Neutrase PF-hydrolyzed fillet		ABTS: 131.80 µmol TE/g FRAP: 3.03 mg AAE/g Peroxyl: 13.60 µmol TE/g	
**Chemical Assays—Collagen and Gelatin**			
Bromelain-hydrolyzed skin collagen	*Pangasius pangasius*	Concentration tested: NR DPPH: 61.67% Reducing power: A_700_ = 0.41	[11]
Bromelain-hydrolyzed bone collagen		Concentration tested: NR DPPH: 71.83% Reducing power: A_700_ = 0.38	
Visceral alkaline protease-hydrolyzed skin gelatin (GTH) Trypsin-hydrolyzed skin gelatin (CTH) Izyme AL^®^-hydrolyzed skin gelatin (IZH)	*Pangasianodon gigas*	ABTS: IZH > CTH > GTH FRAP: CTH > GTH > IZH Ferrous chelating: IZH > CTH > GTH All hydrolysates showed significantly higher antioxidant activity than non-hydrolyzed gelatin control.	[64]
Visceral peptidase-hydrolyzed gelatin		At 5 mg protein/mL: DPPH: 14.69 µmol TE/g protein ABTS: 176.24 µmol TE/g protein FRAP: 38.84 µmol TE/g protein Ferrous chelating: 5.43 mmol EDTA/g protein	[69]
Trypsin-hydrolyzed gelatin		At 5 mg protein/mL: DPPH: 10.80 µmol TE/g protein ABTS: 113.27 µmol TE/g protein FRAP: 26.68 µmol TE/g protein Ferrous chelating: 9.93 mmol EDTA/g protein	
Flavourzyme-hydrolyzed bone gelatin	*P. hypophthalmus*	DPPH: IC_50_ 1.87 mg/mL	[66]
Sonication-treated swim bladder collagen	*Pangasius* sp.	ABTS: IC_50_ 0.044 mg/mL	[72]
Collagenase + trypsin-hydrolyzed skin collagen	Species not specified	At 2 mg/mL: DPPH: 74.31% Reducing power: A_700_ = 0.382	[63]
**Chemical Assays—Viscera, Roe and Multi-Tissue By-Products**			
Alcalase-hydrolyzed roe (1st stage, Fragment 2)	*Nemapteryx caelata*	At 1 mg/mL: DPPH: 84.2% FRAP: 111.3 µmol Fe^2+^/mg Ferrous chelating: 79.2%	[65]
Alcalase-hydrolyzed roe (2nd stage, Fragment 2)		At 1 mg/mL: DPPH: 28.25% FRAP: 193.7 µmol Fe^2+^/mg Ferrous chelating: 50.0%	
Alcalase-hydrolyzed abdominal cut-off isolate	*P. hypophthalmus*	Concentration tested: NR DPPH scavenging: 86.1% Total Reducing Power Capacity: 6.4 mg VCE/g	[20]
Alcalase-hydrolyzed catfish by-product (<1 kDa fraction)		DPPH: IC_50_ 1.31 mg/mL FRAP: 906.90 µM TE	[21]
Papain-hydrolyzed viscera	*Pangasius* sp.	DPPH (2 mg/mL): 65.75% ABTS (3.5 mg/mL): 80.55% Reducing power (5 mg/mL): A_700_ = 0.47	[13]
Pepsin-hydrolyzed viscera		DPPH (2 mg/mL): 90.87% ABTS (3.5 mg/mL): 88.33% Reducing power (5 mg/mL): A_700_ = 0.84	
**GI Digestion Model**			
INFOGEST digest of steamed meat	*Ictalurus punctatus*	At 10 mg/mL: DPPH: 31.35%; ABTS: 64.79% Ferrous chelating: 86.69%	[70]
Pepsin-pancreatin digest of peptide fraction	*Clarias macrocephalus* × *C. gariepinus*	DPPH reduced by ~50% after duodenal digestion	[16]
**In vivo Models**			
Alcalase-hydrolyzed flesh	*Tachysurus sinensis*	Model: HFD-induced obesity mice; dosage: 500 mg/kg Liver MDA reduced to 16.63 nM/mg (vs. HFD control: 31.14 nM/mg) Liver GSH increased to 30.65 μM/mg (vs. HFD control: 10.99 μM/mg) Liver CAT increased to 22.56 U/mg (vs. HFD control: 10.33 U/mg) Liver SOD increased to 2.29 U/mg (vs. HFD control: 0.87 U/mg)	[73]
**Food Systems**			
Alcalase-hydrolyzed muscle in shortfin scad/surimi emulsion sausage	*Pangasius* sp.	All formulations contained 3% hydrolysate Lowest PV and TBARS levels achieved by 100% surimi formulation.	[68]
Alcalase-hydrolyzed muscle in shortfin scad emulsion sausage		3% hydrolysate reduced PV and TBARS over 12-day storage vs. control	[12]

Note: “At X mg/mL” indicates all listed assays were performed at that concentration; “Assay (X mg/mL): value” denotes assay-specific concentrations; IC_50_/EC_50_ represent potency (concentration required for 50% effect). Abbreviations: AAE, ascorbic acid equivalent; ABTS, 2,2′-azino-bis(3-ethylbenzothiazoline-6-sulfonic acid); CAT, catalase; DH, degree of hydrolysis; DPPH, 2,2-diphenyl-1-picrylhydrazyl; EDTA, ethylenediaminetetraacetic acid; FRAP, ferric reducing antioxidant power; GI, gastrointestinal; GSH, glutathione; HFD, high-fat diet; MDA, malondialdehyde; NR, not reported; PV, peroxide value; SOD, superoxide dismutase; TBARSs, thiobarbituric acid reactive substances; TEs, Trolox equivalents; VCE, vitamin C equivalent.

## Data Availability

No new data were created or analyzed in this study. Data sharing is not applicable to this article.

## Abbreviations

The following abbreviations are used in this manuscript:

AAE	Ascorbic acid equivalent
ABTS	2,2′-azino-bis(3-ethylbenzothiazoline-6-sulfonic acid)
CAT	Catalase
DH	Degree of hydrolysis
DPPH	2,2-diphenyl-1-picrylhydrazyl
EC_50_	Half maximal effective concentration
EDTA	Ethylenediaminetetraacetic acid
ET	Electron transfer
E/S	Enzyme/substrate ratio
FRAP	Ferric reducing antioxidant power
GI	Gastrointestinal
GSH	Glutathione
HAT	Hydrogen atom transfer
HFD	High-fat diet
IC_50_	Half maximal inhibitory concentration
MDA	Malondialdehyde
MW	Molecular weight
ORAC	Oxygen radical absorbance capacity
PV	Peroxide value
ROS	Reactive oxygen species
RSM	Response surface methodology
SAR	Structure–activity relationship
SOD	Superoxide dismutase
TBARSs	Thiobarbituric acid reactive substances
TEs	Trolox equivalents
VCE	Vitamin C equivalent
